# First-line treatments for BCG-naïve non-muscle invasive bladder cancer: a systematic review and meta-analysis

**DOI:** 10.1007/s00345-025-06180-5

**Published:** 2026-01-05

**Authors:** Navid Roessler, Brigida A. Maiorano, Marcin Miszczyk, Keiichiro Miyajima, Shota Inoue, Markus von Deimling, Malte W. Vetterlein, Margit Fisch, Marco Moschini, Pawel Rajwa, Andrea Necchi, Morgan Roupret, Benjamin Pradere, David D’Andrea, Paolo Gontero, Pierre I. Karakiewicz, Shahrokh F. Shariat

**Affiliations:** 1 Department of Urology, Comprehensive Cancer Center, Medical University of Vienna, Vienna, Austria; 2 Department of Urology, Medical University Center Hamburg-Eppendorf, Martinistraße 52, 20246 Hamburg, Germany; 3 Department of Medical Oncology, IRCCS San Raffaele Hospital, Milan, Italy; 4 Collegium Medicum, Faculty of Medicine, WSB University, Dąbrowa Górnicza, Poland; 5 Department of Urology, The Jikei University School of Medicine, Tokyo, Japan; 6 Department of Urology, Dentistry and Pharmaceutical Sciences, Okayama University Graduate School of Medicine, Okayama, Japan; 7 Department of Urology, San Raffaele Hospital and Scientific Institute, Milan, Italy; 8 Department of Urology, Medical University of Silesia, Zabrze, Poland; 9 Vita-Salute San Raffaele University, Milan, Italy; 10 GRC 5 Predictive Onco-Uro, Department of Urology, AP-HP, Pitié Salpétrière Hospital, Sorbonne University, Paris, France; 11 Department of Urology UROSUD, La Croix du Sud Hôpital, Quint Fonsegrives, France; 12 Department of Urology, University of Torino School of Medicine, Città della Salute e della Scienza, Torino, Italy; 13 Cancer Prognostics and Health Outcomes Unit, Division of Urology, University of Montréal Health Center, Montréal, QC H2X 0A9 Canada; 14 Department of Urology, University of Texas Southwestern, Dallas, TX USA; 15 Hourani Center for Applied Scientific Research, Al-Ahliyya Amman University, Amman, Jordan; 16 Karl Landsteiner Institute of Urology and Andrology, Vienna, Austria; 17 Research Center for Evidence Medicine, Urology Department, Tabriz University of Medical Sciences, Tabriz, Iran

**Keywords:** Urinary bladder neoplasms, Bacillus calmette guérin, Immune checkpoint inhibitors, Immunotherapy, Treatment outcome

## Abstract

**Purpose:**

To evaluate novel intravesical and systemic combination therapies for improving outcomes in BCG-naïve patients with non-muscle invasive bladder cancer (NMIBC), this systematic review and meta-analysis assessed first-line treatment strategies, including combinations with systemic immune-checkpoint inhibitors (ICIs).

**Methods:**

In this prospectively registered review (CRD420251163026), MEDLINE, Embase, Web of Science, and the ESMO 2025 abstract book were searched for randomized controlled trials (RCTs) evaluating first-line therapies in BCG-naïve NMIBC. Meta-analyses estimated HRs for recurrence-related time-to-event outcomes (disease-free and event-free survival). Grade ≥ 3 treatment-related adverse events were pooled as relative risks (RRs). Risk of bias was assessed using the Cochrane RoB 2 tool.

**Results:**

Out of 5202 records screened, six RCTs including 3485 patients were eligible. Modifications to intravesical BCG alone (three trials, *n* = 895) did not significantly improve recurrence-related outcomes. Systemic ICIs given additional to intravesical BCG significantly reduced recurrence-related events compared to BCG alone (HR 0.77, 95%CI 0.6–0.97, *n* = 1899; number needed to treat at two years: 25), but increased grade ≥ 3 treatment-related adverse events by a statistically and clinically significant margin (RR 3.97, 95% CI 2.53–6.22, *n* = 1879; number needed to treat to harm one patient: 5). Sensitivity analysis using the ALBAN alternative endpoint of high-grade recurrence-free survival yielded HR 0.78 (95% CI 0.59–1.02), not reaching statistical significance. Limitations included heterogeneity in trial design and endpoint definitions.

**Conclusion:**

In BCG-naïve NMIBC, systemic ICIs combined with intravesical BCG improve recurrence-related outcomes but are offset by a substantially increased risk of severe treatment-related adverse events. These findings highlight the need for careful, risk-based, biomarker-guided patient selection to balance over- and undertreatment within a shared decision-making process.

**Supplementary Information:**

The online version contains supplementary material available at 10.1007/s00345-025-06180-5.

## Introduction

Standard of care for patients with high-risk (HR) non-muscle invasive bladder cancer (NMIBC) is intravesical Bacillus Calmette-Guérin (BCG) [1]. While BCG treatment results in initial responses in up 70% of patients, approximately 30–40% of patients with HRNMIBC experience disease recurrence and/or progression within 2–3 years, compounded by the high rate of treatment discontinuation due to treatment-related adverse events [2, 3]. These limitations have prompted the development and evaluation of strategies such as addition of systemic immune-checkpoint inhibitors (ICIs) or intravesical chemotherapy [4].

ICIs, alone or in combination, are used across different disease stages of advanced bladder cancer (BCa), including the perioperative [5–8], BCG-unresponsive [9], and metastatic settings [10]. Preclinical evidence suggests a potential synergy when combining ICIs with intravesical BCG in HRNMIBC patients [11, 12]. These and other strategies have recently been reported in randomized controlled trials (RCTs). This systematic review and meta-analysis of RCTs aims to assess the statistical, clinical, and oncologic efficacy, as well as the tolerability, of adding therapies to intravesical BCG or replacing BCG in BCG-naïve HRNMIBC patients following transurethral resection of bladder tumor (TURBT).

## Materials and methods

**Fig. 1:**
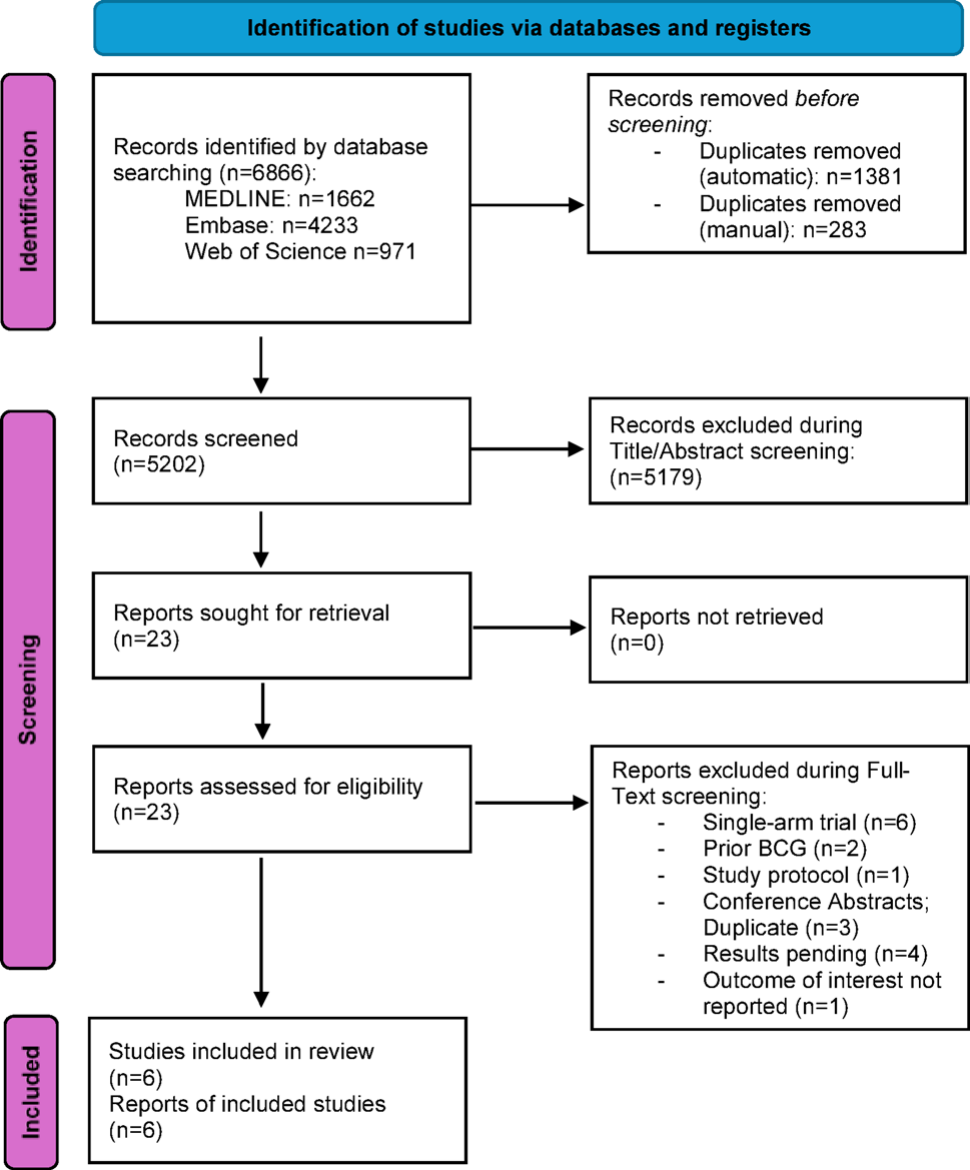
Preferred reporting items for systematic reviews and meta-analyses (PRISMA) - flow diagram. Source: Page MJ, et al. BMJ 2021;372:n71. doi: 10.1136/bmj.n71. This work is licensed under CC BY 4.0. To view a copy of this license, visit https://creativecommons.org/licenses/by/4.0/

This systematic review and meta-analysis was registered with the International Prospective Register of Systematic Reviews (CRD420251163026) and conducted adhering to the Preferred Reporting Items for Systematic Reviews and Meta-analyses (PRISMA) flowchart/checklist (Fig. 1, Supplementary File 8), the AMSTAR2 checklist (Supplementary File 9), and recent guidelines for systematic reviews and meta-analyses [13–15]. Ethical approval and consent to participate were not applicable; no specific funding was received for this work.

### Study selection

The research question and inclusion criteria were defined using the population, intervention, comparison, outcome, and study design (PICOS) framework (Supplementary File 7). We searched MEDLINE (via PubMed), Embase, the Web of Science Core Collection, and the ESMO 2025 abstract book for studies evaluating patients with NMIBC who received first-line intravesical therapy following TURBT. We included RCTs (phase II and phase III) assessing BCG-naïve populations and comparing BCG-based regimens, BCG in combination with systemic or other agents, or alternative intravesical chemotherapy protocols. Both full-text publications, conference abstracts, and trial registries were considered. Studies limited to single-arm designs, non-randomized cohorts, or observational analyses were excluded to ensure population and methodological homogeneity. Non-English manuscripts, reviews, editorials, and studies without original data were also excluded.

The search strategy was performed in October 2025, and the detailed search strategy is provided in Supplementary File 2. Reports were merged and de-duplicated using EndNoteX9 (Clarivate); the title-abstract screening was conducted independently by two authors followed by full-text assessment. Backward citation searching was performed to identify potentially relevant additional records. At each step of the review, conflicts were resolved through consensus among co-authors.

### Data extraction

Two authors independently extracted data, including the first author’s name, year of publication, trial name and trial period, patient characteristics (e.g., age, sex), tumor characteristics (e.g., T-stage), safety data (e.g., number of grade ≥ 3 adverse events [AEs]), median follow-up and outcomes of interest, including recurrence-related time-to-event endpoints such as disease-free survival (DFS), event-free survival (EFS), recurrence-free survival (RFS), and overall survival (OS). All conflicts that arose during the data extraction process were resolved through discussion between authors.

### Risk of bias assessment

Each study was evaluated independently by two authors using the Cochrane Collaboration’s Risk-of-Bias assessment tool version 2.0 (RoB2) for randomized-controlled trials (RCTs) [16].

### Statistical analysis

Random-effects meta-analyses were conducted to estimate hazard ratios (HRs) for recurrence-related time-to-event outcomes (defined as DFS and EFS), among first-line intravesical and combination systemic therapies in BCG-naïve HRNMIBC. For treatment-related AEs, random-effects models were applied to estimate relative risks (RRs) for all and treatment-related grade ≥ 3 events. The absolute risk increase (ARI) for severe adverse events was calculated from the pooled event rates in the intervention and control groups, and the number needed to harm (NNH) was derived as the inverse of this ARI. Similarly, the absolute risk reduction (ARR) for recurrence-related outcomes at two years was calculated from the event-free or disease-free survival rates in the intervention and control arms, with the number needed to treat (NNT) defined as the inverse of the ARR. When 2-year recurrence-related events were not reported, the corresponding Kaplan–Meier curves were digitised using WebPlotDigitizer software (version 4.6.0) to extract the necessary data [17]. HRs were log-transformed and standard errors calculated from reported confidence intervals. Between-study heterogeneity was assessed both qualitatively and quantitatively using *I*², which was estimated with the DerSimonian-Laird method within a random-effects model. Forest-plots were generated to visualize individual trial estimates and pooled effect sizes. All analyses were two-sided and performed in R version 4.4.2, R Foundation for Statistical Computing, Vienna, Austria).

## Results

Out of 5202 individual records screened, six RCTs comprising 3485 patients were included for this systematic review and meta-analysis [18–23]. Among these, five trials were phase III trials (*n* = 3435), and one was a phase II trial (*n* = 50) [20]. All trials focused on patients with HRNMIBC. The most investigated intervention was intravesical BCG therapy, often compared to combination therapies with systemic ICIs. Across the included RCTs, the median/mean age ranged from 67 to 74 years, with most patients being male. T1 tumours accounted for 44% to 62% of patients across the individual study arms, while carcinoma in situ (CIS) was present in 12% to 39% of patients. Median follow-up durations varied between 12 and 61 months. Adverse events of grade ≥ 3 were documented in five trials [18, 20–23], ranging from 4% to 29% across the individual study arms. Table 1 summarizes the characteristics of the included RCTs.

**Table 1:** Demographics and clinical characteristics of the included randomized-controlled trials

Author	Trial name; period; phase	Randomization	Age, years	No. of patients	Male gender, *n* (%)	Tumor stage, *n* (%)	CIS, *n* (%)	Adverse events ≥ grade 3, *n* (%)	Median follow-up, months	Clinical endpoint (treatment vs. control), HR (95%CI)
Grimm, M. O., et al. (2020)	NIMBUS; Dec 2013 to Oct 2019, Phase-III	Standard frequency BCG (15 instillations in total)	NR	175	146 (83)	T0: 3 (1.7); Ta: 74 (42); T1: 98 (56);	48 (27)	NR	12 (safety analysis)	Time to recurrence: HR 0.4 (0.24–0.68)
		Reduced frequency BCG (9 instillations in total)		170	138 (81)	Ta: 82 (48); T1: 87 (51); > T1: 1 (0.6)	44 (26)			
Guerrero-Ramos, F., et al. (2022)	HIVEC-HR; Nov 2016 to March 2021; Phase-II	Hyperthermic intravesical chemotherapy (IND and MNT)	mean 74 (SD +/- 10)	25	21 (84)	Ta: 14 (56); T1: 11 (44)	NR	4 (16)	33.7 (IQR 18.6–37.1)	RFS: HR 0.47 (0.13–1.61); PFS: HR 0.14 (0.02–1.19); OS: HR 0.75 (0.23–2.48)
		BCG (IND and MNT)	mean 73 (SD +/- 8.7)	25	22 (88)	Ta: 11 (44); T1: 14 (56)		6 (24)		
Hayne, D., et al. (2025)	ANZUP 1301; Dec 2013 to May 2023; Phase-III	BCG (IND and MNT) + mitomycin (given weeks 3, 6, and 9 and months 3, 6, and 9)	median 70 (IQR 63–77)	248	NR	Ta: 265 (53); T1: 235 (47)	140 (28)	43 (17)	47 (IQR 31–64)	DFS: HR 0.86 (0.64–1.14); RFS: HR 0.84 (0.61–1.18); PFS: HR 0.74 (0.45–1.21); OS: HR 1.07 (0.61–1.88)
		BCG (IND and MNT)		252				37 (15)		
Shore, N. D., et al. (2025)	CREST; Jan 2020 to Nov 2021; Phase-III	Sasanlimab + BCG (IND and MNT) [Arm A]	median 67 (range 31–91)	352	280 (80)	Ta: 96 (27); T1: 204 (58)	52 (15)	102 (29)	36.3 (EFS analysis)	EFS (Arm A vs. C): HR 0.68 (0.49–0.94); EFS (Arm B vs. C): HR 1.16 (0.87–1.55); OS (Arm A vs. C): HR 1.13 (0.68–1.87); OS (Arm B vs. C): HR 1.07 (0.64–1.79)
		Sasanlimab + BCG (IND) [Arm B]		352	299 (85)	Ta: 136 (39); T1: 174 (49)	42 (12)	76 (22)		
		BCG (IND and MNT) [Arm C]		351	284 (81)	Ta: 107 (31); T1: 194 (55)	50 (14)	22 (6.3)		
Roupret, M., et al. (2025)	ALBAN; Dec 2018 to Jan 2025; Phase-III	BCG (IND and MNT)	median 67 (range 29–91)	255	439 (85)	Ta: 110 (21); T1: 204 (40)	202 (39)	22 (8.8)	35.3 (range 0–60)	EFS: HR 0.98 (0.71–1.36) HG-RFS: HR 1.06 (0.72–1.54)
		Atezolizumab + BCG (IND and MNT)		262				58 (23)		
De Santis, M., et al. (2025)	POTOMAC; June 2018 to Oct 2020; Phase-III	Durvalumab + BCG (IND and MNT) [Arm A]	median 68 (range 24–90)	339	276 (81)	Ta: 112 (33); T1: 195 (58)	125 (37)	71 (21)	60.7 (IQR 51.5–665)	DFS (Arm A vs. C): HR 0.68 (0.5–0.93); DFS (Arm B vs. C): HR 1.14 (0.86–1.5); OS (Arm A vs. C): HR 0.8 (0.53–1.2)
		Durvalumab + BCG (IND) [Arm B]	median 68 (range 21–87)	339	272 (80)	Ta: 114 (34); T1: 191 (56)	125 (37)	52 (15)		
		BCG (IND and MNT) [Arm C]	median 67 (range 32–86)	340	271 (80)	Ta: 99 (29); T1: 211 (62)	125 (37)	13 (4)		

AE = Adverse event; BCG = Bacillus Calmette–Guérin; CIS = Carcinoma in situ; CI = Confidence interval; DFS = Disease-free survival; EFS = Event-free survival; HG-RFS = High-grade recurrence-free survival; HR = Hazard ratio; IND = Induction; IQR = Interquartile range; MNT = Maintenance; NR = Not reported; OS = Overall survival; PFS = Progression-free survival; RFS = Recurrence-free survival

Percentages may not add up to 100%, as they are rounded.

### Assessment of risk of bias

The risk of bias judgments of each domain for each included study are summarised in Supplementary File 3. According to the RoB 2 tool for RCTs, three trials were rated as low risk of bias [18, 20, 23], two as some concerns due to missing outcome data and incomplete reporting in non–peer-reviewed abstracts [21, 22], and one as high risk of bias owing to early trial termination [19], which limits interpretability.

### Interventions and clinical outcomes of the included randomized controlled trials

Three of the included RCTs evaluated alternative intravesical approaches (*n* = 895, one reducing BCG intensity, one adding intravesical mitomycin [MMC] to BCG, and one replacing BCG with externally heated MMC) [19–21], while the other three investigated adding systemic ICIs to intravesical BCG (*n* = 2590) [18, 22, 23].

#### Intravesical therapy modifications

In the NIMBUS trial (*n* = 345), reduced-frequency intravesical BCG (RF-BCG; induction: weeks 1, 2, and 6; maintenance: weeks 1 and 3 at months 3, 6, and 12; nine instillations total) was compared with standard-frequency BCG (SF-BCG; induction: weeks 1–6; maintenance: weeks 1–3 at months 3, 6, and 12; 15 instillations total). RF-BCG showed significantly inferior outcomes for time to first recurrence (HR 0.4, 95% CI 0.24–0.68), leading to early termination of the trial [19]. 

In the ANZUP 1301 trial (*n* = 500), patients were randomized to intravesical BCG + MMC (BCG + MMC) or BCG alone. The BCG + MMC arm received alternating weekly instillations of BCG (weeks 1, 2, 4, 5, 7, 8) and MMC (weeks 3, 6, 9) during induction, followed by maintenance every four weeks for nine cycles (BCG: weeks 21, 33, 45; MMC: weeks 13, 17, 25, 29, 37, 41; total = 9 BCG + 6 MMC doses). The BCG-alone arm received weekly BCG for six weeks (induction) and four-weekly maintenance for ten cycles (16 BCG doses total). No significant difference was observed in 2-year DFS (76% vs. 71%; HR 0.86, 95% CI 0.64–1.14) or RFS (81% vs. 75%; HR 0.84, 95% CI 0.61–1.18) [21]. 

The HIVEC-HR trial (*n* = 50) compared hyperthermic intravesical chemotherapy with MMC (HIVEC-MMC) to intravesical BCG. HIVEC consisted of weekly instillations for six weeks (induction) and monthly instillations for six months (maintenance), starting 40 days postoperatively. BCG was given weekly for six weeks (induction) and weekly for three weeks at months 3, 6, and 12 (maintenance). The primary endpoint, RFS, showed no statistically significant difference between groups (HR 0.47, 95% CI 0.13–1.61) [20]. 

#### Systemic ICI combinations with intravesical BCG

The CREST trial (*n* = 1055) evaluated systemic sasanlimab plus intravesical BCG induction and maintenance (Arm A), sasanlimab plus BCG induction only (Arm B), and BCG induction + maintenance (Arm C). In Arm A, sasanlimab was administered subcutaneously every four weeks for up to 25 cycles, combined with BCG induction and maintenance (median duration 98 weeks, 18 doses). Arm B received sasanlimab plus BCG induction only (median duration six weeks, six doses), and Arm C received BCG induction and maintenance (median duration 99 weeks, 21 doses). The primary endpoint, investigator-assessed EFS (time to high-grade recurrence, progression, CIS persistence, or death), showed improvement in Arm A vs. Arm C (HR 0.68, 95% CI 0.49–0.94), but not between Arm B and Arm C (HR 1.16, 95% CI 0.87–1.55) [23]. 

The ALBAN trial (*n* = 517) compared intravesical BCG (six-weekly induction, three-weekly maintenance at months 3, 6, and 12; Arm A) with systemic atezolizumab (1200 mg IV every three weeks for up to one year) plus BCG as in Arm A (Arm B). The primary endpoint, EFS (time to recurrence, progression, CIS persistence, upper-tract UC, or death), showed no significant difference between arms (HR 0.98, 95% CI 0.71–1.36) [22]. 

The POTOMAC trial (*n* = 1018) assessed durvalumab (1500 mg IV every four weeks for 13 cycles) combined with BCG induction and two years of maintenance (Arm A), durvalumab plus BCG induction only (Arm B), and BCG induction + maintenance (Arm C). The primary endpoint, DFS (time to high-grade recurrence, progression, CIS persistence, or death), demonstrated a significant benefit for Arm A vs. Arm C (HR 0.68, 95% CI 0.50–0.93), while Arm B showed no difference (HR 1.14, 95% CI 0.86–1.50) [18]. 

### Meta-analysis: recurrence-related time to event for intravesical BCG combined with systemic ICIs

**Fig. 2:**
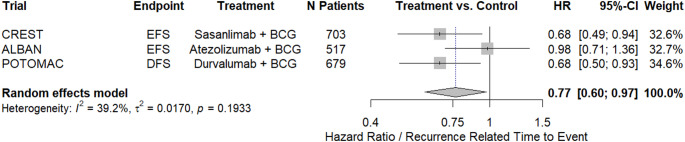
Forest-Plot: Meta-analysis of recurrence-related time to event for intravesical BCG combined with systemic immunotherapy

For the meta-analysis, three RCTs evaluating a combination of systemic ICI with intravesical BCG (induction and maintenance) versus intravesical BCG alone (induction and maintenance) were included [18, 22, 23]. The respective primary endpoints were EFS for CREST and ALBAN, and DFS for POTOMAC, which were considered comparable for the purposes of recurrence-related time-to-event analysis. In a pooled analysis (*n* = 1899), the combination therapy was associated with a statistically significant reduction in the risk of recurrence-related events compared with BCG alone (HR 0.77, 95%CI: 0.6–0.97; Fig. 2). There was evidence of substantial heterogeneity among the included RCTs (*I*²=39%), primarily driven by the ALBAN trial, which demonstrated lower efficacy and because EFS included low grade recurrence, which CREST and POTOMAC did not consider as an event [18, 22, 23].

A sensitivity analysis was performed excluding the ALBAN trial as the main source of heterogeneity [22]. In this sensitivity analysis (*n* = 1382), the combination therapy remained statistically significantly beneficial (HR 0.68, 95%CI: 0.54–0.85; Supplementary File 4), without evidence of substantial heterogeneity among the included RCTs.

### Sensitivity analysis: recurrence-related time to event using an alternative endpoint definition

A sensitivity analysis was conducted to account for differences in the recurrence-related endpoint definition in the ALBAN trial [22]. Using high-grade RFS, defined as reappearance of high-grade NMIBC relaps or death from any cause, in a pooled analysis (*n* = 1899) [18, 19, 22], the combination therapy showed a similar reduction in the risk of recurrence-related events compared with BCG alone (HR 0.78, 95%CI: 0.59–1.02; Supplementary File 5), although this did not reach level of statistical significance. There was evidence of substantial heterogeneity among the included RCTs (*I*²=49%), primarily driven by the ALBAN trial.

### Treatment-related adverse-events among the included randomized controlled trials

Five RCTs (*n* = 2429) reported data on treatment-related AEs [18, 20–23]. In the ANZUP 1301 trial, grade ≥ 3 AEs occurred in 17% of patients receiving BCG plus mitomycin compared with 15% in patients receiving BCG alone, with fatigue, renal/urinary disorders, and flu-like symptoms being the most frequently reported events [21]. In the HIVEC-HR trial, 65% of patients reported at least one AE, and 48% reported at least one treatment-related AE. Grade ≥ 3 events were slightly more frequent in the BCG arm (24% vs. 16%), including one treatment-related death [20]. 

In the CREST trial, treatment-related AEs of any grade occurred in 87%, 79%, and 70% of patients in the sasanlimab plus BCG induction and maintenance, sasanlimab plus BCG induction only, and BCG alone arms, respectively. Grade ≥ 3 AEs were observed in 29%, 22%, and 6.3% of patients across these arms, with dysuria, hematuria, and elevated lipase among the most common severe events [23]. For the ALBAN trial, grade ≥ 3 AEs occurred in 8.8% of patients receiving BCG alone and 23% in the atezolizumab plus BCG arm, most commonly cystitis or urinary tract disorders [22]. In the POTOMAC trial, severe treatment-related AEs were reported in 21%, 15%, and 4% of patients receiving durvalumab plus BCG induction and maintenance, durvalumab plus BCG induction only, and BCG alone, respectively, with dysuria being the most frequent AE. Immune-mediated AEs were observed primarily in patients receiving durvalumab, with hypothyroidism, hepatitis, and rash as the most common events; no treatment-related deaths were reported [18]. 

### Meta-analysis: treatment-related severe adverse events for systemic ICIs combined with intravesical BCG

**Fig. 3:**
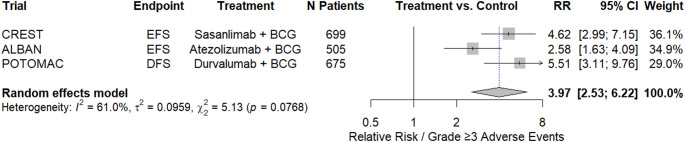
Forest-Plot: meta-analysis of treatment-related severe adverse events for systemic immunotherapy combined with intravesical BCG

Three RCTs reporting grade ≥ 3 treatment-related adverse events were included in the meta-analysis [18, 22, 23]. The pooled meta-analysis (*n* = 1879) demonstrated a statistically significant increase in the risk of severe treatment-related adverse events for patients receiving systemic ICIs compared to intravesical BCG compared with intravesical BCG alone (RR 3.97, 95%CI: 2.53–6.22; Fig. 3). There was evidence of moderate heterogeneity across the included studies (*I*²=61%).

### Meta-analysis: number needed to harm and number needed to treat for systemic ICIs combined with intravesical BCG

Across the included RCTs reporting grade ≥ 3 treatment-related events from systemic ICIs combined with intravesical BCG [18, 19, 22], the absolute risk of severe adverse events was 25.8% in the combination therapy group versus 6.5% with BCG alone, corresponding to a NNH of five, indicating that for every five patients treated with the combination therapy, one additional patient experienced a severe treatment-related adverse event.

For recurrence-related outcomes at two years, the NNT was calculated based on the 2-year event-free or disease-free survival reported in the individual trials (Supplementary File 5). Considering all three trials (CREST, ALBAN, POTOMAC; *n* = 1899) [18, 19, 22], the pooled absolute risk reduction was 3.7%, resulting in a pooled NNT of 25, meaning that treating 25 patients with combination therapy would result in one additional patient remaining recurrence-free at two years. Given the heterogeneity in outcome definitions observed in the ALBAN trial, a sensitivity analysis including only CREST and POTOMAC (*n* = 1382) yielded a pooled NNT of 20, meaning that treating 20 patients with combination therapy would result in one additional patient remaining recurrence-free at two years.

## Discussion

In this systematic review and meta-analysis, we synthesized data from RCTs in BCG-naïve patients with HRNMIBC. In a pooled analysis of three trials evaluating systemic ICIs in combination with intravesical BCG, the addition of systemic ICIs was associated with a statistically significant reduction in recurrence-related events compared with BCG alone. However, this benefit must be weighed against an increased risk of severe treatment-related adverse events, underscoring the need to balance efficacy and safety when considering combination strategies.

Modifications to BCG administration alone have failed to maintain efficacy, as exemplified by the NIMBUS trial [19], where a reduced-frequency BCG schedule was terminated early due to clear inferiority in time-to-recurrence compared with standard-frequency BCG. Similarly, the ANZUP 1301 trial [21], comparing BCG plus MMC to BCG alone, did not demonstrate improvements in recurrence-related outcomes or safety, reaffirming standard BCG as the reference treatment. Replacing BCG with extracorporeally heated MMC also failed to improve RFS compared with BCG [20]. Full-dose BCG induction followed by maintenance remains the cornerstone of effective therapy, and systemic ICIs cannot be considered a replacement for BCG maintenance. Contemporary outcomes with modern TURBT and adequate BCG demonstrate markedly improved 3-year EFS across all patients compared to historical benchmarks [24–26]. Notably, the addition of durvalumab for one year or sasanlimab for 2 years in HR patients improved the chance of remaining free from HR recurrence at 3 years by 5–7%, although at the cost of a 20–30% risk of treatment related severe adverse events. Despite this toxicity, no clinically meaningful deterioration in OS was observed, suggesting that the increased rate of severe adverse events did not compromise long-term survival outcomes. As bladder preservation remains a key therapeutic priority in NMIBC, the balance between incremental efficacy in delaying recurrence and the potential for severe toxicity is critical when evaluating treatment intensification or combination strategies.

Heterogeneity in trial design and endpoint definitions complicates cross-trial comparisons, with the ALBAN trial standing somewhat apart from CREST and POTOMAC [18, 22, 23]. The ALBAN trial differed fundamentally in its treatment schedule, duration of ICI exposure, and primary endpoint definition. Whereas CREST and POTOMAC implemented full BCG maintenance schedules with ICIs administered during both induction and maintenance phases [18, 23], ALBAN limited atezolizumab to an abbreviated BCG maintenance at months 3, 6, and 12 [22]. Correspondingly, the median number of BCG instillations was substantially lower in ALBAN (12 cycles) compared to CREST (18 cycles) and POTOMAC (20 cycles), a reduction reminiscent of the NIMBUS trial, where decreased BCG intensity was associated with inferior recurrence control and led to premature termination [19].

Median follow-up durations were similar between ALBAN and CREST, though longer in POTOMAC, potentially contributing to differences in observed outcomes, specifically AEs [18, 22, 23]. Notably, ALBAN also enrolled the highest proportion of patients with CIS, introducing additional heterogeneity in risk composition. Potentially, the choice of ICI inhibitor may have influenced results. Atezolizumab has previously shown limited perioperative benefit in muscle-invasive bladder cancer, as demonstrated in the IMvigor010 trial [7], suggesting that its efficacy profile may differ from PD-1–targeting agents such as sasanlimab (CREST) or durvalumab (POTOMAC). The rapid evolution of ICIs scheduling, the introduction of novel bispecific agents, and the development of device-assisted delivery approaches collectively position NMIBC treatment as a moving target.

To date, none of the systemic ICIs evaluated in combination with intravesical BCG (durvalumab, sasanlimab, or atezolizumab) have received regulatory approval for the treatment of BCG-naïve high-risk NMIBC. Although two of the three phase III trials (POTOMAC and CREST) demonstrated a significant improvement in recurrence-related outcomes with prolonged ICI exposure alongside full-dose BCG maintenance [18, 23], the substantial increase in grade ≥ 3 treatment-related adverse events raises concerns regarding the overall risk-benefit ratio. These trials were never designed to replace BCG, but rather to explore whether intensification with systemic ICIs could further reduce high-grade recurrence rates in selected patients. Given the availability of subsequent therapeutic options, broad adoption of these combinations appears premature. Future approval and clinical implementation should be limited to carefully selected patients, particularly those with high-risk features who are strongly committed to bladder preservation and fully informed about the substantial risk of severe treatment-related adverse events. Ideally, selection should be guided by a robust shared decision-making process that prioritizes patient-centered outcomes. Pending health-related quality of life data will be critical in assessing whether the toxicity profile outweighs the oncologic benefits and in informing any broader clinical implementation.

The findings of this meta-analysis should be interpreted considering several limitations. First, not all anticipated trial results were available at the time of analysis (October 2025), with key data from phase II/III studies like SUNRISE-3, BRIDGE, and QUILT-2.005 still pending, potentially altering pooled estimates as the evidence base matures [27–29]. Second, substantial clinical and methodological heterogeneity precluded granular subgroup analyses (e.g., by tumor stage, ICI class, or risk group such as very-HR NMIBC), driven by variations in BCG strains, endpoint selection, and patient cohorts (e.g., the POTOMAC trial included patients with multiple, recurrent, or large tumors ≥ 3 cm). Due to substantial heterogeneity in intervention types and comparator arms among the included trials, a broader network meta-analysis was not feasible; consequently, our meta-analysis was restricted to trials evaluating systemic ICIs in combination with intravesical BCG. Digitisation of the ALBAN trial Kaplan–Meier curves with WebPlotDigitizer software may have introduced inaccuracies. Evidence of substantial heterogeneity in our meta-analysis, primarily driven by the ALBAN trial, likely reflects differences in outcome definitions, as ALBAN included low-grade recurrences and upper-tract urothelial carcinoma in its event-free survival endpoint. Nevertheless, excluding this trial from analyses did not alter the findings by a significant degree.

## Conclusion

Intensification of treatment with systemic ICIs in combination with intravesical BCG reduces high-grade recurrence rates in patients with HRNMIBC. However, this benefit is counterbalanced by a substantial increase in severe treatment-related adverse events, emphasizing the need to carefully weighing efficacy against toxicity. Critical questions remain regarding optimal endpoint selection, patient-centered outcomes, and the identification of those most likely to benefit from treatment intensification, underscoring that biomarker driven and/or risk-based patient selection will be central to translating therapeutic advances into meaningful clinical benefit while minimizing toxicity and preserving quality of life.

## Supplementary Information

Below is the link to the electronic supplementary material.

Supplementary Material 1

## Data Availability

No datasets were generated or analysed during the current study.
