# Supplementary material for: First-line treatments for BCG-naïve non-muscle invasive bladder cancer: a systematic review and meta-analysis
Source: World J Urol. 2026 Jan 5;44(1):76. doi: 10.1007/s00345-025-06180-5 (PMC12769649; doi:10.1007/s00345-025-06180-5)

Supplementary File

[**Supplementary File 1 - Preferred Reporting Items for Systematic Reviews and Meta-analyses (PRISMA) - flow diagram for new systematic reviews which included searches of databases and registers only** 2](#_Toc213777113)

[**Supplementary File 2 - Detailed Search Strategy for the databases** 3](#_Toc213777114)

[**Supplementary File 3: Risk of Bias according to Cochrane Collaboration’s Risk-of-Bias assessment tool version 2.0 (RoB2)** 7](#_Toc213777115)

[**Supplementary File 4: Sensitivity Analysis - Meta-Analysis: Recurrence-Related Time-to-Event for Intravesical BCG Combined with Systemic Immunotherapy Excluding the Main Source of Heterogeneity** 8](#_Toc213777116)

[**Supplementary File 5: Sensitivity Analysis - Meta-Analysis: Recurrence-related Time to Event Using an Alternative Endpoint Definition** 9](#_Toc213777117)

[**Supplementary File 6: Meta-Analysis – Number Needed to Treat for Systemic Immunotherapy Combined with Intravesical BCG** 10](#_Toc213777118)

[**Supplementary File 7: PICO(S) Framework** 11](#_Toc213777119)

[**Supplementary File 8: PRISMA Checklist** 12](#_Toc213777120)

[**Supplementary File 9: AMSTAR 2 Checklist** 15](#_Toc213777121)

# **Supplementary File 1 - Preferred Reporting Items for Systematic Reviews and Meta-analyses (PRISMA) - flow diagram for new systematic reviews which included searches of databases and registers only**

Studies included in review

(n=6)

Reports of included studies

(n=6)

**Identification of studies via databases and registers**

**Screening**

Records screened

(n=5202)

Records excluded during Title/Abstract screening:

(n=5179)

Reports sought for retrieval

(n=23)

Reports not retrieved

(n=0)

Reports assessed for eligibility

(n=23)

Reports excluded during Full-Text screening:

- Single-arm trial (n=6)
- Prior BCG (n=2)
- Study protocol (n=1)
- Conference Abstracts; Duplicate (n=3)
- Results pending (n=4)
- Outcome of interest not reported (n=1)

**Included**

**Identification**

Records identified by database searching (n=6866):

MEDLINE: n=1662

Embase: n=4233

Web of Science n=971

Records removed *before screening*:

- Duplicates removed (automatic): n*=*1381
- Duplicates removed (manual): n=283

Source: Page MJ, et al. BMJ 2021;372:n71. doi: 10.1136/bmj.n71.

This work is licensed under CC BY 4.0. To view a copy of this license, visit <https://creativecommons.org/licenses/by/4.0/>

# **Supplementary File 2 - Detailed Search Strategy for the databases**

MEDLINE (via PubMed) – 10/2025

| #1 | (  "non-muscle invasive bladder cancer"[Title/Abstract]  OR "nonmuscle invasive bladder cancer"[Title/Abstract]  OR "NMIBC"[Title/Abstract]  OR "superficial bladder cancer"[Title/Abstract]  OR "Ta"[Title/Abstract]  OR "T1"[Title/Abstract]  OR "carcinoma in situ"[Title/Abstract]  OR "CIS"[Title/Abstract]  OR "Urinary Bladder Neoplasms"[MeSH Terms]  ) | 440344 |
| --- | --- | --- |
| #2 | (  ("BCG"[Title/Abstract]  OR "Bacillus Calmette-Guérin"[MeSH Terms]  OR "intravesical therapy"[Title/Abstract]  OR "intravesical administration"[Title/Abstract]  OR "drug therapy"[MeSH Terms])  OR ("chemotherapy"[Title/Abstract]  OR "immunotherapy"[Title/Abstract]  OR "systemic therapy"[Title/Abstract]  OR "intravenous"[Title/Abstract]  OR "subcutaneous"[Title/Abstract])  OR ("gemcitabine"[Title/Abstract]  OR "docetaxel"[Title/Abstract]  OR "mitomycin"[Title/Abstract]  OR "epirubicin"[Title/Abstract]  OR "valrubicin"[Title/Abstract])  OR ("PD-1 inhibitor"[Title/Abstract]  OR "PD-L1 inhibitor"[Title/Abstract]  OR "checkpoint inhibitor"[Title/Abstract]  OR "durvalumab"[Title/Abstract]  OR "atezolizumab"[Title/Abstract]  OR "sasanlimab"[Title/Abstract])  OR ("gene therapy"[Title/Abstract]  OR "viral vector"[Title/Abstract]  OR "oncolytic virus"[Title/Abstract]  OR "adenoviral therapy"[Title/Abstract]  OR "nadofaragene firadenovec"[Title/Abstract]  OR "cretostimogene grenadenorepvec"[Title/Abstract]  OR "TAR-200"[Title/Abstract]  OR "drug delivery system"[Title/Abstract]  OR "sustained release"[Title/Abstract])  OR ("N803"[Title/Abstract]  OR "nogapendekin alfa inbakicept"[Title/Abstract]  OR "IL-15 agonist"[Title/Abstract])  ) | 2528980 |
| #3 | (  "BCG-naïve"[Title/Abstract]  OR "BCG naive"[Title/Abstract]  OR "no prior BCG"[Title/Abstract]  OR "first-line"[Title/Abstract]  OR "initial treatment"[Title/Abstract]  OR "BCG-unexposed"[Title/Abstract]  ) | 161060 |
| #4 | #1 AND #2 AND #3 | **1662** |

Embase – 10/2025

| #1 | (  'non-muscle invasive bladder cancer':ti,ab  OR 'nonmuscle invasive bladder cancer':ti,ab  OR 'NMIBC':ti,ab  OR 'superficial bladder cancer':ti,ab  OR 'Ta':ti,ab  OR 'T1':ti,ab  OR 'carcinoma in situ':ti,ab  OR 'CIS':ti,ab  OR 'urinary bladder neoplasms'/exp  ) | 586756 |
| --- | --- | --- |
| #2 | (  ('BCG':ti,ab  OR 'bacillus calmette-guerin'/exp  OR 'intravesical therapy':ti,ab  OR 'intravesical administration':ti,ab  OR 'drug therapy'/exp)  OR ('chemotherapy':ti,ab  OR 'immunotherapy':ti,ab  OR 'systemic therapy':ti,ab  OR 'intravenous':ti,ab  OR 'subcutaneous':ti,ab)  OR ('gemcitabine':ti,ab  OR 'docetaxel':ti,ab  OR 'mitomycin':ti,ab  OR 'epirubicin':ti,ab  OR 'valrubicin':ti,ab)  OR ('PD-1 inhibitor':ti,ab  OR 'PD-L1 inhibitor':ti,ab  OR 'checkpoint inhibitor':ti,ab  OR 'durvalumab':ti,ab  OR 'atezolizumab':ti,ab  OR 'sasanlimab':ti,ab)  OR ('gene therapy':ti,ab  OR 'viral vector':ti,ab  OR 'oncolytic virus':ti,ab  OR 'adenoviral therapy':ti,ab  OR 'nadofaragene firadenovec':ti,ab  OR 'cretostimogene grenadenorepvec':ti,ab  OR 'TAR-200':ti,ab  OR 'drug delivery system':ti,ab  OR 'sustained release':ti,ab)  OR ('N803':ti,ab  OR 'nogapendekin alfa inbakicept':ti,ab  OR 'IL-15 agonist':ti,ab)  ) | 5543057 |
| #3 | (  'BCG-naive':ti,ab  OR 'BCG naïve':ti,ab  OR 'no prior BCG':ti,ab  OR 'first-line':ti,ab  OR 'initial treatment':ti,ab  OR 'BCG-unexposed':ti,ab  ) | 287829 |
| #4 | #1 AND #2 AND #3 | **4233** |

Web of Science – 10/2025

| #1 | TS=("non-muscle invasive bladder cancer"  OR "nonmuscle invasive bladder cancer"  OR "NMIBC"  OR "superficial bladder cancer"  OR "Ta"  OR "T1"  OR "carcinoma in situ"  OR "CIS"  OR "urinary bladder neoplasms")  AND  TS=("BCG"  OR "Bacillus Calmette-Guérin"  OR "intravesical therapy"  OR "intravesical administration"  OR "drug therapy"  OR "chemotherapy"  OR "immunotherapy"  OR "systemic therapy"  OR "intravenous"  OR "subcutaneous"  OR "gemcitabine"  OR "docetaxel"  OR "mitomycin"  OR "epirubicin"  OR "valrubicin"  OR "PD-1 inhibitor"  OR "PD-L1 inhibitor"  OR "checkpoint inhibitor"  OR "durvalumab"  OR "atezolizumab"  OR "sasanlimab"  OR "gene therapy"  OR "viral vector"  OR "oncolytic virus"  OR "adenoviral therapy"  OR "nadofaragene firadenovec"  OR "cretostimogene grenadenorepvec"  OR "TAR-200"  OR "drug delivery system"  OR "sustained release"  OR "N803"  OR "nogapendekin alfa inbakicept"  OR "IL-15 agonist")  AND  TS=("BCG-naïve"  OR "BCG naive"  OR "no prior BCG"  OR "first-line"  OR "initial treatment"  OR "BCG-unexposed") | **971** |
| --- | --- | --- |

# **Supplementary File 3: Risk of Bias according to Cochrane Collaboration’s Risk-of-Bias assessment tool version 2.0 (RoB2)**


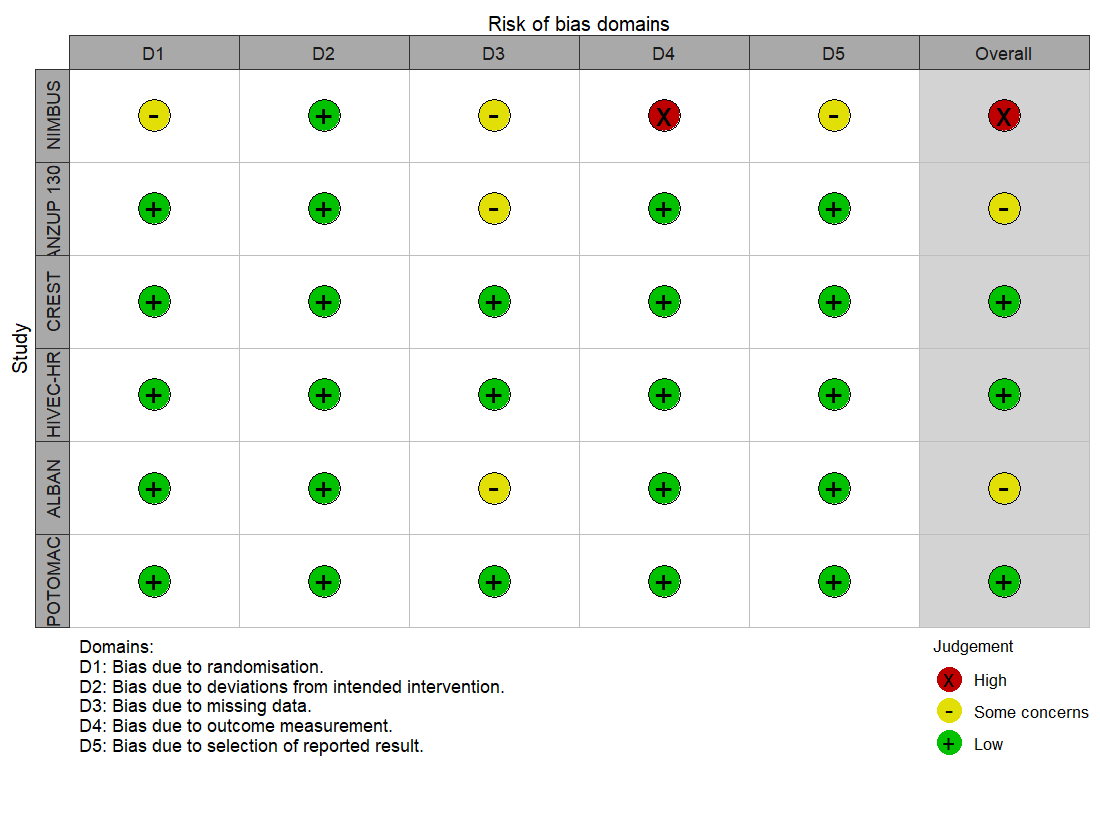


#
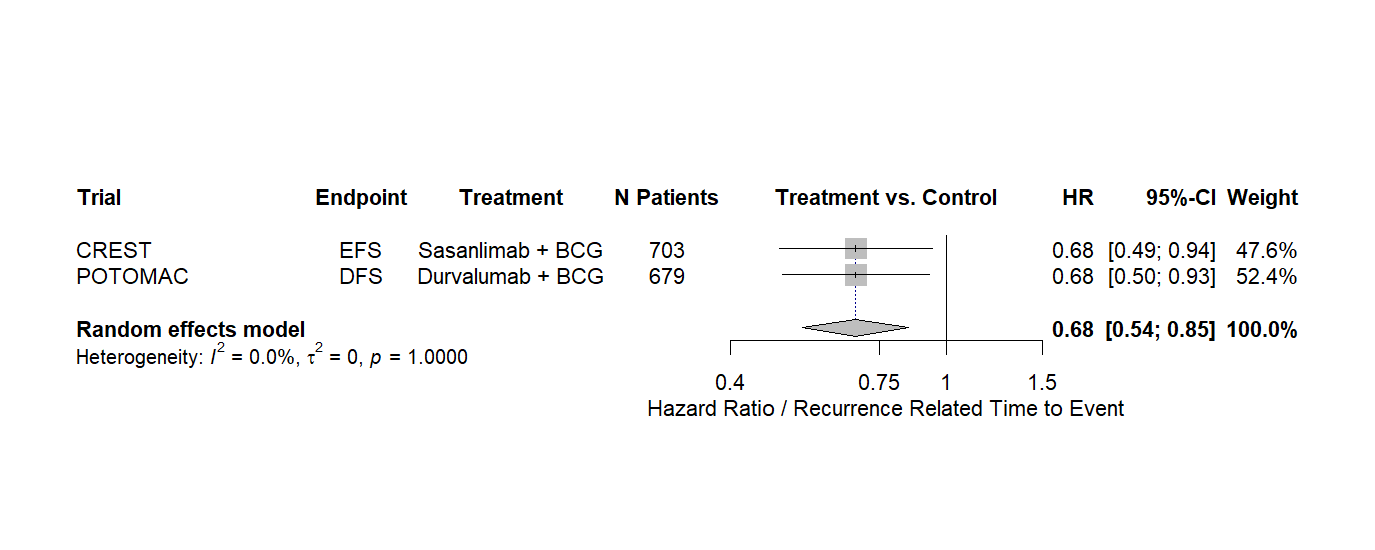
**Supplementary File 4: Sensitivity Analysis - Meta-Analysis: Recurrence-Related Time-to-Event for Intravesical BCG Combined with Systemic Immunotherapy Excluding the Main Source of Heterogeneity**

# **Supplementary File 5: Sensitivity Analysis - Meta-Analysis: Recurrence-related Time to Event Using an Alternative Endpoint Definition**


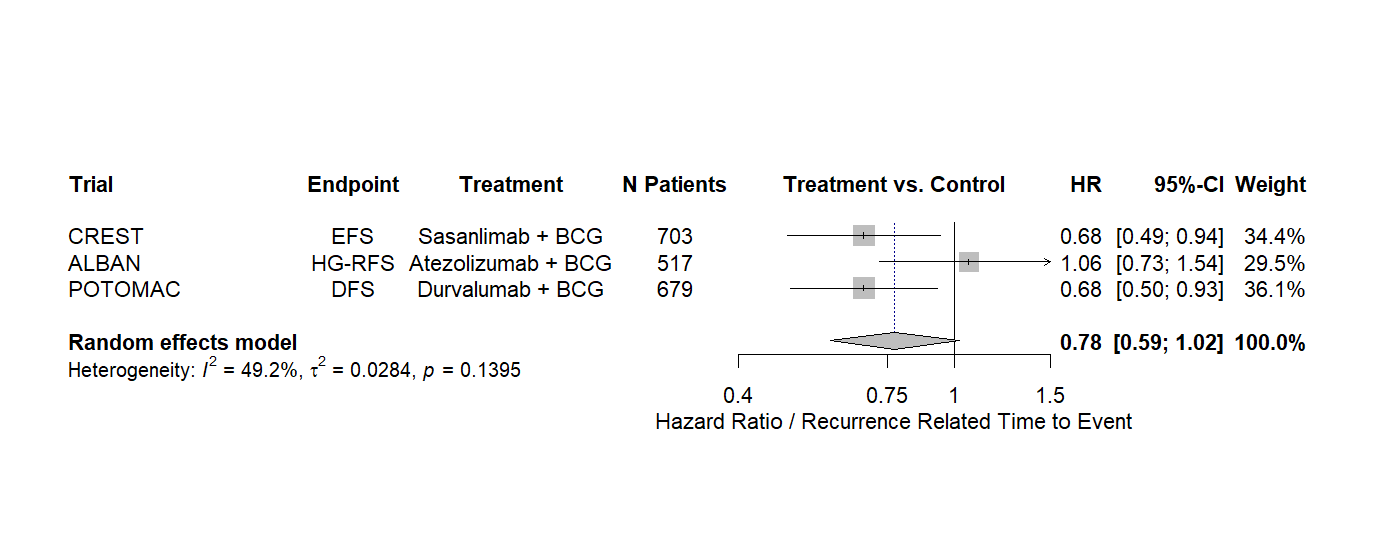


# **Supplementary File 6: Meta-Analysis – Number Needed to Treat for Systemic Immunotherapy Combined with Intravesical BCG**

Two-Year Recurrence-Related Outcomes and Numbers at Risk Across Trials

| Trial | Recurrence-Related Endpoint (%) at 2 years (Immunotherapy + BCG); Number at risk | Recurrence-Related Endpoint (%) at 2 years (BCG); Number at risk | Absolute Risk Reduction | Number Needed to Treat |
| --- | --- | --- | --- | --- |
| CREST | EFS: 84.7%; n=241 | EFS: 79.9%; n=240 | 4.89% | n=21 |
| ALBAN | EFS: 75.3%; n=157 | EFS: 74.4%; n=155 | 0.68% | n=142 |
| POTOMAC | DFS: 86.5%; n=262 | DFS: 81.6%; n=258 | 4.96% | n=20 |

# **Supplementary File 7: PICO(S) Framework**

| **Component** | **Description** |
| --- | --- |
| **Population (P)** | Adults (≥18 years) with histologically confirmed, high- or intermediate-risk non–muscle-invasive bladder cancer who are Bacillus Calmette–Guérin (BCG)–naïve and have undergone complete transurethral resection of bladder tumor (TURBT) |
| **Intervention (I)** | First-line intravesical therapies administered after TURBT, including BCG (monotherapy or in combination with perivascular or other agents) or alternative intravesical chemotherapy regimens or novel agents. |
| **Comparator (C)** | Standard intravesical BCG therapy alone, placebo, or no adjuvant intravesical treatment. |
| **Outcomes (O)** | **Primary:** Recurrence related time to event (Event-free survival, Disease-free survival, Recurrence-free survival) and overall survival (OS).  **Secondary:** Safety and tolerability (treatment-related adverse events) **Exploratory:** Treatment ranking and relative efficacy assessed through network meta-analysis. |
| **Study Design (S)** | Randomized controlled trials (RCTs; phase II or III) and subsequent/post-hoc analyses. Conference abstracts and trial registries were considered for completeness. |

# **Supplementary File 8: PRISMA Checklist**

| **Section and Topic** | **Item #** | **Checklist item** | **Location where item is reported** |
| --- | --- | --- | --- |
| **TITLE** | | |  |
| Title | 1 | Identify the report as a systematic review. | Title/Abstract |
| **ABSTRACT** | | |  |
| Abstract | 2 | See the PRISMA 2020 for Abstracts checklist. | Abstract |
| **INTRODUCTION** | | |  |
| Rationale | 3 | Describe the rationale for the review in the context of existing knowledge. | Introduction |
| Objectives | 4 | Provide an explicit statement of the objective(s) or question(s) the review addresses. | Introduction |
| **METHODS** | | |  |
| Eligibility criteria | 5 | Specify the inclusion and exclusion criteria for the review and how studies were grouped for the syntheses. | Study selection (2.1.) |
| Information sources | 6 | Specify all databases, registers, websites, organisations, reference lists and other sources searched or consulted to identify studies. Specify the date when each source was last searched or consulted. | Study selection (2.1) |
| Search strategy | 7 | Present the full search strategies for all databases, registers and websites, including any filters and limits used. | Supplementary File 2 |
| Selection process | 8 | Specify the methods used to decide whether a study met the inclusion criteria of the review, including how many reviewers screened each record and each report retrieved, whether they worked independently, and if applicable, details of automation tools used in the process. | Study selection (2.1.) |
| Data collection process | 9 | Specify the methods used to collect data from reports, including how many reviewers collected data from each report, whether they worked independently, any processes for obtaining or confirming data from study investigators, and if applicable, details of automation tools used in the process. | Data extraction (2.2.) |
| Data items | 10a | List and define all outcomes for which data were sought. Specify whether all results that were compatible with each outcome domain in each study were sought (e.g. for all measures, time points, analyses), and if not, the methods used to decide which results to collect. | Data extraction (2.2.) |
|  | 10b | List and define all other variables for which data were sought (e.g. participant and intervention characteristics, funding sources). Describe any assumptions made about any missing or unclear information. | Data extraction (2.2.) |
| Study risk of bias assessment | 11 | Specify the methods used to assess risk of bias in the included studies, including details of the tool(s) used, how many reviewers assessed each study and whether they worked independently, and if applicable, details of automation tools used in the process. | Risk of bias assessment (2.3.) |
| Effect measures | 12 | Specify for each outcome the effect measure(s) (e.g. risk ratio, mean difference) used in the synthesis or presentation of results. | Statistical Analysis (2.4.) |
| Synthesis methods | 13a | Describe the processes used to decide which studies were eligible for each synthesis (e.g. tabulating the study intervention characteristics and comparing against the planned groups for each synthesis (item #5)). | Statistical Analysis (2.4.) |
|  | 13b | Describe any methods required to prepare the data for presentation or synthesis, such as handling of missing summary statistics, or data conversions. | Statistical Analysis (2.4.) |
|  | 13c | Describe any methods used to tabulate or visually display results of individual studies and syntheses. | Statistical Analysis (2.4.) |
|  | 13d | Describe any methods used to synthesize results and provide a rationale for the choice(s). If meta-analysis was performed, describe the model(s), method(s) to identify the presence and extent of statistical heterogeneity, and software package(s) used. | Statistical Analysis (2.4.) |
|  | 13e | Describe any methods used to explore possible causes of heterogeneity among study results (e.g. subgroup analysis, meta-regression). | Statistical Analysis (2.4.) |
|  | 13f | Describe any sensitivity analyses conducted to assess robustness of the synthesized results. | Statistical Analysis (2.4.) |
| Reporting bias assessment | 14 | Describe any methods used to assess risk of bias due to missing results in a synthesis (arising from reporting biases). | Risk of bias assessment (2.3.) |
| Certainty assessment | 15 | Describe any methods used to assess certainty (or confidence) in the body of evidence for an outcome. | Statistical Analysis (2.4.) |
| **RESULTS** | | |  |
| Study selection | 16a | Describe the results of the search and selection process, from the number of records identified in the search to the number of studies included in the review, ideally using a flow diagram. | Results (3.) |
|  | 16b | Cite studies that might appear to meet the inclusion criteria, but which were excluded, and explain why they were excluded. | Assessment of risk of bias (3.1.) |
| Study characteristics | 17 | Cite each included study and present its characteristics. | Results (3.2.) |
| Risk of bias in studies | 18 | Present assessments of risk of bias for each included study. | Assessment of risk of bias (3.1.) |
| Results of individual studies | 19 | For all outcomes, present, for each study: (a) summary statistics for each group (where appropriate) and (b) an effect estimate and its precision (e.g. confidence/credible interval), ideally using structured tables or plots. | Results (3.3., 3.4., 3.5., 3.6., 3.7.) |
| Results of syntheses | 20a | For each synthesis, briefly summarise the characteristics and risk of bias among contributing studies. | Results (3.3., 3.4., 3.5., 3.6., 3.7.) |
|  | 20b | Present results of all statistical syntheses conducted. If meta-analysis was done, present for each the summary estimate and its precision (e.g. confidence/credible interval) and measures of statistical heterogeneity. If comparing groups, describe the direction of the effect. | Results (3.3., 3.4., 3.5., 3.6., 3.7.) |
|  | 20c | Present results of all investigations of possible causes of heterogeneity among study results. | Results (3.3., 3.4., 3.5., 3.6., 3.7.) |
|  | 20d | Present results of all sensitivity analyses conducted to assess the robustness of the synthesized results. | Results (3.3., 3.4., 3.5., 3.6., 3.7.) |
| Reporting biases | 21 | Present assessments of risk of bias due to missing results (arising from reporting biases) for each synthesis assessed. | Assessment of risk of bias (3.1.) |
| Certainty of evidence | 22 | Present assessments of certainty (or confidence) in the body of evidence for each outcome assessed. | Results (3.3., 3.4., 3.5., 3.6., 3.7.) |
| **DISCUSSION** | | |  |
| Discussion | 23a | Provide a general interpretation of the results in the context of other evidence. | Discussion (4.) |
|  | 23b | Discuss any limitations of the evidence included in the review. | Discussion (4.) |
|  | 23c | Discuss any limitations of the review processes used. | Discussion (4.) |
|  | 23d | Discuss implications of the results for practice, policy, and future research. | Discussion/Conclusion (4., 5.) |
| **OTHER INFORMATION** | | |  |
| Registration and protocol | 24a | Provide registration information for the review, including register name and registration number, or state that the review was not registered. | Methods (2.) |
|  | 24b | Indicate where the review protocol can be accessed, or state that a protocol was not prepared. | PROSPERO (2.) |
|  | 24c | Describe and explain any amendments to information provided at registration or in the protocol. | PROSPERO (2.) |
| Support | 25 | Describe sources of financial or non-financial support for the review, and the role of the funders or sponsors in the review. | No funding. |
| Competing interests | 26 | Declare any competing interests of review authors. | COI reported in additional File |
| Availability of data, code and other materials | 27 | Report which of the following are publicly available and where they can be found: template data collection forms; data extracted from included studies; data used for all analyses; analytic code; any other materials used in the review. | All data are fully available within the manuscript |

*From:*  Page MJ, McKenzie JE, Bossuyt PM, Boutron I, Hoffmann TC, Mulrow CD, et al. The PRISMA 2020 statement: an updated guideline for reporting systematic reviews. BMJ 2021;372:n71. doi: 10.1136/bmj.n71. This work is licensed under CC BY 4.0. To view a copy of this license, visit <https://creativecommons.org/licenses/by/4.0/>

# **Supplementary File 9: AMSTAR 2 Checklist**


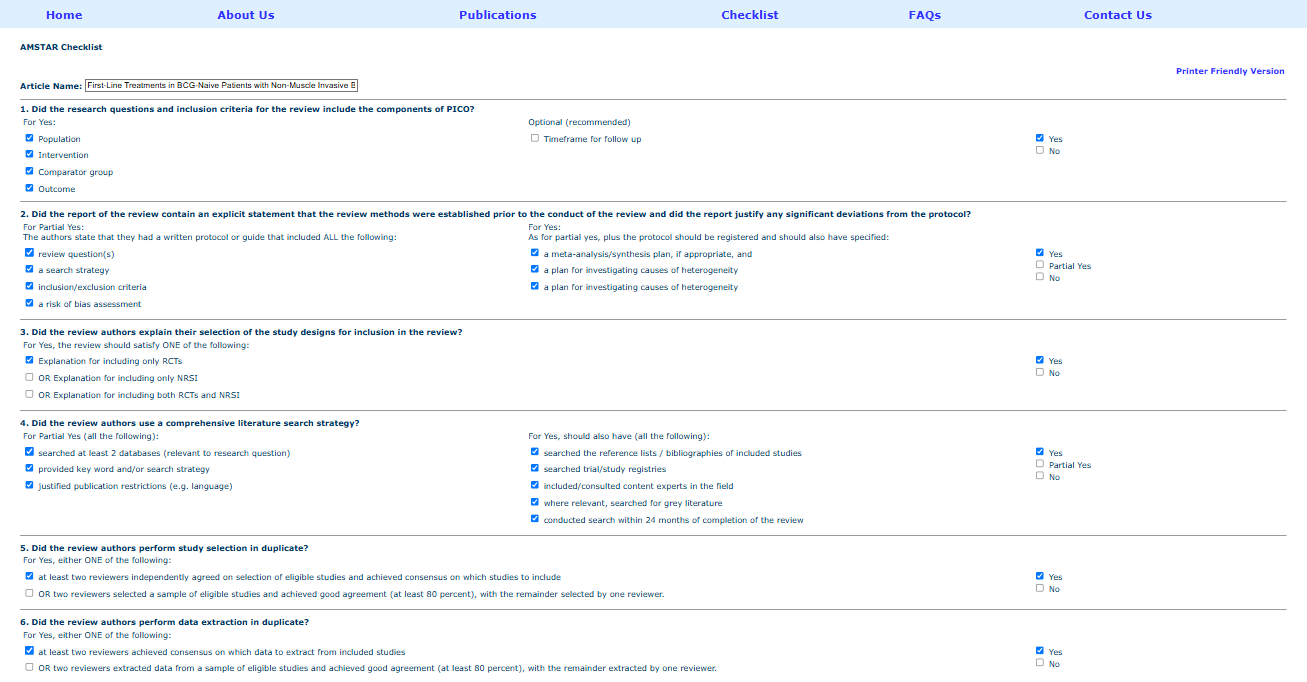

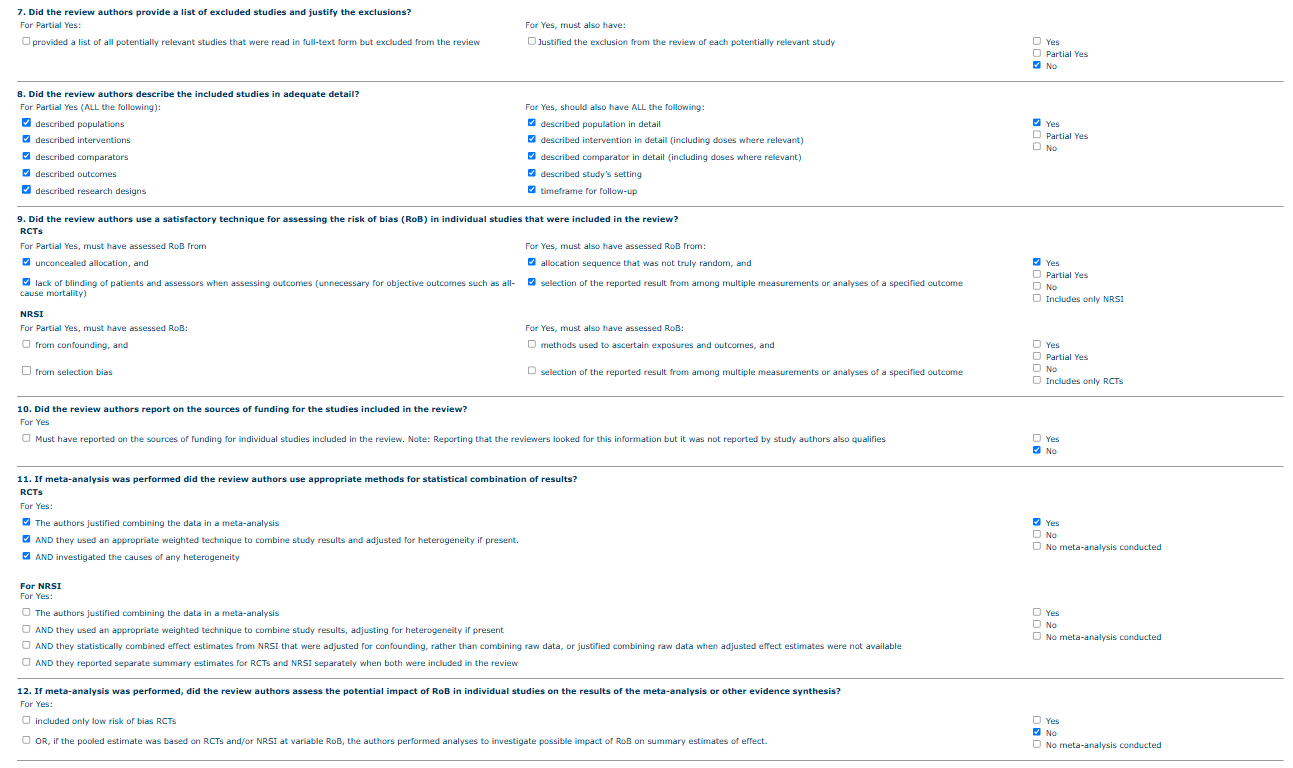

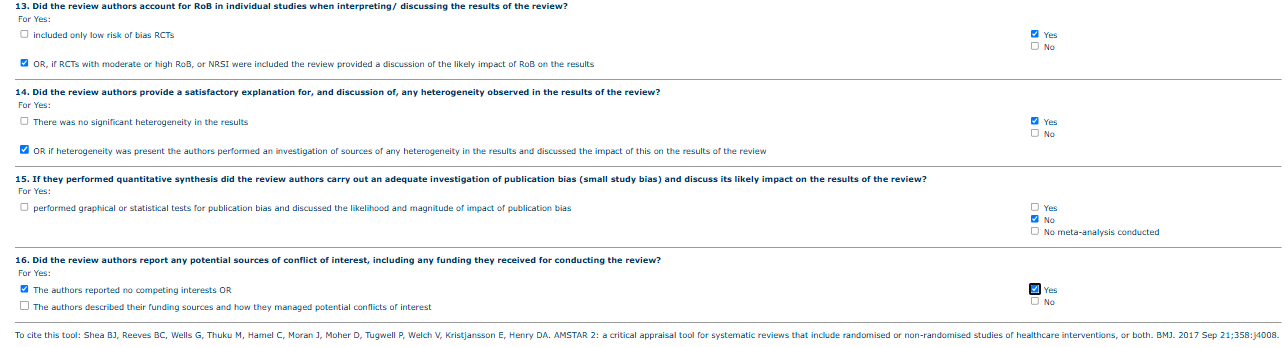

Supplement: Supplementary file 1 — Supplementary Material 1 [file 345_2025_6180_MOESM1_ESM.docx]
